# Ambient Temperature Is Correlated With the Severity of Neonatal Hypoxic-Ischemic Brain Injury via Microglial Accumulation in Mice

**DOI:** 10.3389/fped.2022.883556

**Published:** 2022-05-06

**Authors:** Rika Zen, Tomoya Terashima, Shunichiro Tsuji, Miwako Katagi, Natsuko Ohashi, Yuri Nobuta, Asuka Higuchi, Hirohiko Kanai, Takashi Murakami, Hideto Kojima

**Affiliations:** ^1^Department of Stem Cell Biology and Regenerative Medicine, Shiga University of Medical Science, Otsu, Japan; ^2^Department of Obstetrics and Gynecology, Shiga University of Medical Science, Otsu, Japan

**Keywords:** neonatal, mouse-model, brain injury, hypoxic-ischemic encephalopathy, temperature, behavioral changes, cerebral volume loss, microglia

## Abstract

**Background:**

The pathophysiology of neonatal hypoxic-ischemic encephalopathy (HIE) has been studied in several rodent models to develop novel treatments. Although it is well known that high ambient temperature results in severe HIE, the effect of subtle changes in ambient temperature during a hypoxic-ischemic (HI) insult has not been studied. Therefore, in order to clarify the difference of pathophysiological change among the HIE models due to the influence of small changes in chamber temperature, three-step gradual change of 0.5°C each were prepared in ambient temperature during hypoxic exposure.

**Methods:**

Blood flow in the left common carotid artery (CCA) of neonatal mice was interrupted using bipolar electronic forceps under general and local anesthesia. The mice were subsequently subjected to 10% hypoxic exposure for 50 min at 36.0, 36.5, or 37.0°C. A control group was also included in the study. The size of the striatum and hippocampus and the volume reduction rate of the hemisphere in the section containing them on the ischemic side were evaluated using microtubule associated protein 2 (MAP2) immunostaining. The accumulation of Iba1-positive cells was investigated to assess inflammation. Additionally, rotarod and open-field tests were performed 2 weeks after HI insult to assess its effect on physiological conditions.

**Results:**

MAP2 staining revealed that the higher the temperature during hypoxia, the more severe the volume reduction rate in the hemisphere, striatum, and hippocampus. The number of Iba1-positive cells in the ipsilateral lesion gradually increased with increasing temperature, and there was a significant difference in motor function in the 36.5 and 37.0°C groups compared with the sham group. In the open-field tests, there was a significant decrease in performance in the 37.0°C groups compared with the 36.0°C and sham groups.

**Conclusions:**

Even a small gradual change of 0.5°C produced a significant difference in pathological and behavioral changes and contributed to the accumulation of Iba1-positive cells. The arrangement of ambient temperature is useful for creating a rodent model with the appropriate severity of the targeted neuropsychological symptoms to establish a novel therapy for HIE.

## Introduction

Hypoxic-ischemic encephalopathy (HIE) in newborn babies is a brain injury caused by hypoxia and ischemia that occurs in 1–8 per 1,000 live births (1, 2). It can result in severe and permanent neuropsychological sequelae, such as cerebral palsy, epilepsy, intellectual disability, and learning impairments (3–5). Moreover, there is no curative treatment, and the efficiency remains modest even if therapeutic hypothermia, one of the few effective treatments, reduces the rate of mortality and disability (6, 7). Therefore, to establish a novel therapy, various animal models have been used to examine the pathophysiology of HIE in detail. The most common model for neonatal HIE is the modified Rice-Vannucci model (8).

A combination of unilateral common carotid artery (CCA) ligation and hypoxic exposure is used in the modified Rice-Vannucci model (8–12). It has been reported that the HIE model becomes severe when the ambient temperature during hypoxic exposure set to a high temperature (13, 14). However, the influence was only investigated at high temperatures such as 40 or 42°C. It is difficult to determine exactly how a slight change in the ambient temperature during hypoxic exposure affects the severity of pathology and behavioral impairments in HIE because there is a limit to how tightly the temperature can be controlled in the hypoxic chamber. These are critical points both for accurately investigating the pathological condition and for creating a model suitable for the purpose of investigation. The importance of choosing an appropriate animal model according to experimental objectives has been described (15, 16).

Hypoxic-ischemic (HI) insults cause a rapid robust inflammatory response in the neonatal brain (4, 17, 18). These phases constitute an activated apoptotic cascade and are thought to be strongly influenced by microglia, which is a key early factor in the inflammatory response to HI injury (19–21). Controlling microglial inflammation is expected to lead to the development of novel therapies for HIE, such as stem cells, minocycline, and estrogen therapy (22–24). At present, therapeutic hypothermia, which decreases body temperature, is the standard therapy for HIE in clinical settings. It is thought to exert anti-inflammatory effects and shows some benefit in decreasing the rate of mortality and disability (6, 25). In addition, decreased body temperature has an anti-inflammatory effect on microglial activation (26, 27). However, there are no reports investigating the relationship between the precise change in ambient temperature during hypoxic exposure and the activation of microglia.

Here, we focused on the influence of slight changes in chamber temperature during hypoxic exposure on brain injury, which was examined by the preparation of a special chamber that can tightly control the ambient temperature. As the chamber we used is excellent at maintaining the temperature inside, temperature-dependent brain injury could be clarified around the physiologically normal intrauterine temperature range. Three types of chamber temperatures were set to clarify the differences among the HIE models owing to the small change in the ambient temperature. We then investigated how chamber temperature affected cerebral volume loss, pathological changes, and behavioral dysfunction in the HIE model. We also examined the relationship between a small change in ambient temperature during hypoxic exposure and microglial activation.

## Materials and Methods

### Animals

Some sets of three male and three female C57BL/6 neonatal mice with one nursing mother were appropriately purchased from the Jackson laboratory (Bar Harbor, ME, USA) through Charles River Laboratories Japan (Yokohama, Japan) on the second day after birth. After being housed for a week, the mice were used for the following experiments. All mice were bred under a 12-h light-dark cycle with ad libitum access to food and water. In exclusion criteria for animal experiments, mice were planned to exclude from experiments when they showed 5 s or less in the rotarod test, inability to drink or eat, and 20% weight loss within 1 week. However, no mice met these criteria. All experiments comply with the ARRIVE guidelines.

### Hypoxic Chamber

**Figure 1:**
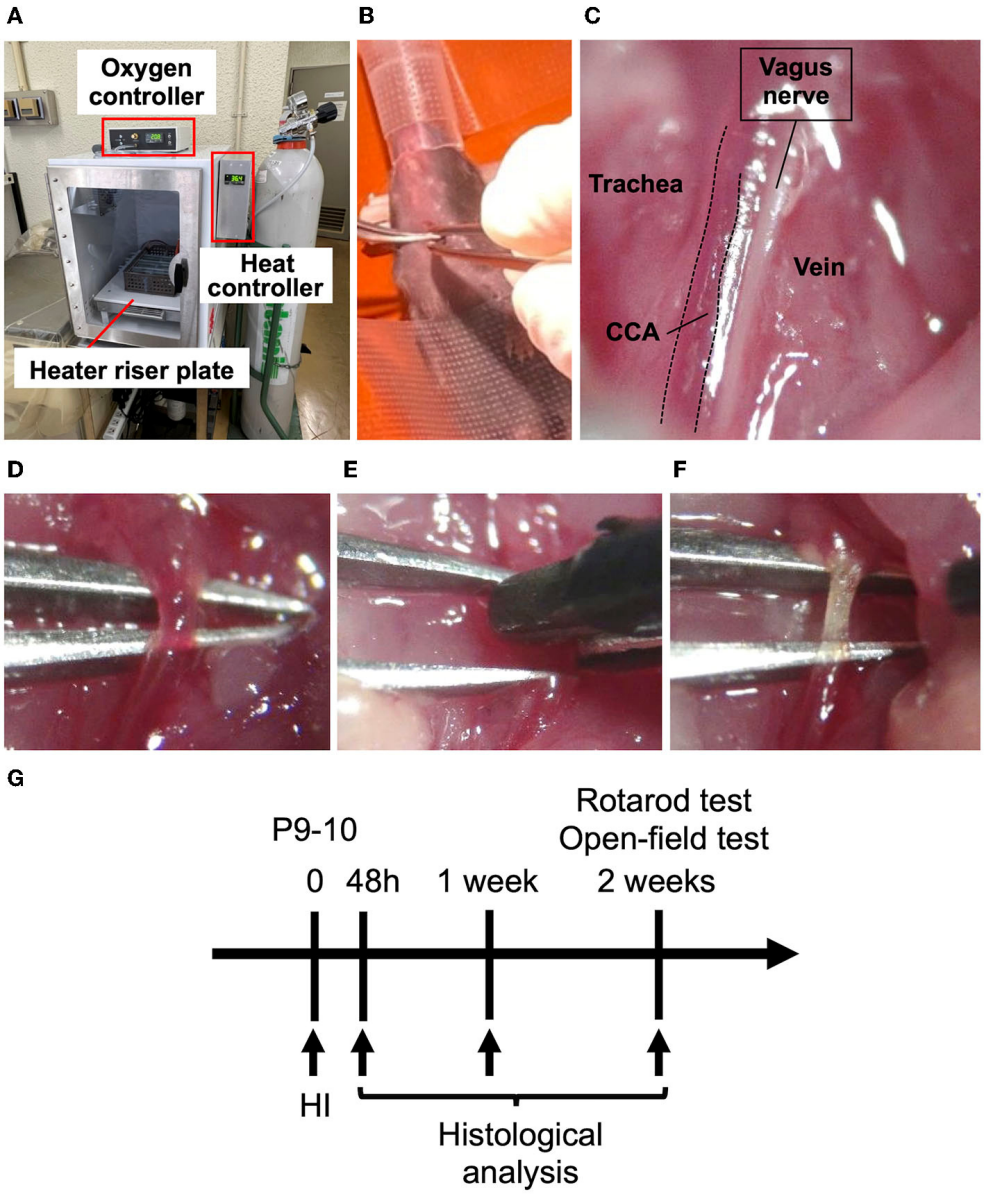
Procedures for creating an HIE neonatal mouse model. **(A)** Hypoxic chamber in which the internal temperature can be controlled. The heater riser plate gets hot and circulates the warm air inside with fans; the ambient temperature is controlled by monitoring the inside temperature using a thermometer on the plate, and is changed by altering the warmth of the plate. The oxygen controller maintains the hypoxia level. **(B)** A P10 mouse placed on its back under sedation with isoflurane. **(C)** Expanding the field of view of the left CCA parallel to the vagus nerve under a stereomicroscope. **(D)** Isolation of the left CCA from the vagus nerve. **(E)** Coagulation of the left CCA by bipolar electronic forceps. **(F)** The left CCA immediately after coagulation. **(G)** The scheme of the timeline for the experiments.

A hypoxic chamber (BioSpherix, Parish, NY, USA) was used to expose the mice to hypoxia (Figure 1A). In this chamber, the oxygen concentration was constantly maintained by an oxygen controller (BioSpherix), and the heater riser plate (BioSpherix) was set to warm up inside. The plate had two exhaust ducts and three fans, which circulated the air and rapidly homogenized the temperature inside. The plate contained a thermometer connected to a heat controller (BioSpherix), which was regulated to automatically adjust to the target temperature.

### Procedure of HI

The HI procedure was performed using a modified protocol based on the Rice-Vannucci model. Male and female mice underwent the HI procedure on postnatal day 9–10 (P9-10) (Figure 1). After sedation with isoflurane (4% for induction and 1.5% for maintenance) and local anesthesia with 0.25% Marcaine (Aspen Japan, Tokyo, Japan), the mice were placed on its back, and the skin was cut on the left cervical. After expanding the field of view of the left neck, the left CCA was isolated and coagulated using bipolar electronic forceps (7 Watt) (Figures 1B–F). The skin incision was ligated by 6-0 silk and infiltrated with 0.25% Marcaine (Aspen) as analgesics. Some pups were dead immediately during and after coagulation of the CCA procedure. However, after recovery from anesthesia, no pups died. The pups were returned to their mother and after 1 h placed in a hypoxic chamber (BioSpherix) with 10% O_2_ in 90% N_2_ for 50 min. We set the pups in the box, which was separated to equal area so that they would not overlap. The ambient temperature was set to 36.0, 36.5, or 37.0°C and monitored every 5 min during hypoxic exposure in the chamber. Body temperature was measured in the axilla of the mice immediately after exposure to hypoxia by using a continuous temperature probe (RET-3; Bio Research Center Co Ltd, Nagoya, Japan) attaching with an automatically temperature-controlled operating bed (BWT-100A; Bio Research Center Co Ltd). In the sham operation, the left CCA was exposed by expanding the field of left neck, but the blood flow was not interrupted by coagulation. The day 9/10-day-old litters of 36.0°C, 36.5°C, 37.0°C and sham groups were used, respectively, sixteen pups (nine P9 and seven P10) from four mothers, fourteen (eight P9 and six P10) from three, thirteen (seven P9 and six P10) from three and twelve (six P9 and six P10) from ten. Control mice were prepared litter mates from each temperature groups as sham groups. The mortality rate after hypoxic exposure was zero %.

### Immunohistostaining

At 48 h, 1 or 2 weeks after HIE generation (Figure 1G), brain tissues were isolated after systemic transcardial perfusion with 4% paraformaldehyde in 0.1 M phosphate buffer. The brains were isolated and embedded in paraffin blocks and sectioned into 5 μm thick slices using a YAMATO microtome at two positions for each brain, corresponding to bregma +0.6 mm, −1.8 mm of adult mouse brain. After deparaffinization, antigen activation was performed using HistoVT One (pH 7.0; Nacalai). After blocking, the sections were incubated with mouse anti-microtubule-associated protein 2 (MAP2) antibody (#M4403, Sigma-Aldrich, St. Louis, MO, USA) or rabbit anti-Iba1 antibody (#019-19741, Wako, Osaka, Japan) as primary antibodies at 4°C overnight for immunohistochemistry. The sections were then incubated with secondary antibody (anti-mouse IgG antibody or anti-rabbit IgG antibody), and were colored using an ABC kit (Vector Laboratories, Burlingame, Canada) and 3,3'-diaminobenzidine (DAKO, Glostrup, Denmark) according to the manufacturer's protocol. The stained cells were observed under a light microscope (IX83; Olympus, Tokyo, Japan) and their pictures were taken by CCD digital microscope camera (DP80; Olympus). For quantification of volume reduction in brain, the pictures in whole brain area were prepared by tiling the over twenty pictures of MAP2-staining sections using cellSens Dimension Software system (Olympus). In the brain sections at bregma +0.6 mm or −1.8 mm, the area of whole ipsilateral hemisphere was compared to that of contralateral side in two slices per each mouse by using imageJ software version 1.51 (NIH, Bethesda, MD, USA). In addition, the area of the striatum or hippocampus on the ipsilateral side was compared to that on the contralateral side in two slices per each mouse by imageJ (NIH). To assess brain inflammation quantitatively, we counted the number of Iba1-positive cells at five separate area in the size of 100 × 100 μm square and calculated the average of them in the striatum section. In the hippocampus section, we counted the number of all Iba1-positive cells in whole hippocampus area. And we divided them by whole hippocampus area. These numbers were compared between the ipsilateral and contralateral sides.

### Rotarod Test

Motor function was analyzed using the rotarod test (Ugo Basile, Comerio-Varese, Italy) 2 weeks after HIE generation (Figure 1G), following previous day's practice as previously described (28). Rotarod tests were performed for 5 min at a speed range from 5 to 50 rpm/min at an acceleration of 9 rpm/min^2^ and time was measured until the mouse dropped. The average of three medians of five trials was calculated for each mouse and used for analysis. All tests were performed in blind to a conductor about the information of which groups the mice belong to.

### Open-Field Test

Emotional behavior was analyzed using an open-field test 2 weeks after HIE generation (Figure 1G). An open-field test was performed for 5 min in a 50 × 50 cm area. Locomotion data were recorded and analyzed using a video tracking system (Muromachi Kikai, Tokyo, Japan). The total distance, mean velocity, and central area time were measured and compared between the sham, 36.0, 36.5, and 37.0°C groups. All tests were performed in blind to a conductor about the information of which groups the mice belong to.

### Classification of Severity in Histological and Behavioral Changes

The severity of the histological damage was classified into five stages according to the grade of cerebral volume reduction. The results in sham group were identified as (–). Those were compared to those in 36.0°C, those in 36.0°C were compared to those in 36.5°C, and those in 36.5°C were compared to those in 37.0°C groups. In each comparison, (+) was added to more severe group from the grade of the comparison group if the group showed < 0.05 in *p*-value, (++) was added to more severe group from the grade of the comparison group if the group showed < 0.01 in *p*-value, and nothing was added if no significant difference. After all, the grades were expressed as +, ++, +++ and ++++. The severity of behavioral disorders was classified by a significant difference compared with other groups: not significant (n.s.) = –, ^*^p < 0.05 = +, ^**^p < 0.01 = ++.

### Statistical Analysis

Data are shown as mean ± SD by using GraphPad Prism software version 6 (GraphPad Software Inc., San Diego, CA, USA). All data were analyzed that they showed normal distribution pattern or not by the Shapiro-Wilk normality test in advance. After confirming the normal distribution, *t*-test was used for the comparison between the two groups as parametric analysis. For multiple datasets, one-way ANOVA was used and followed by the Scheffe's test as a post-hoc parametric analysis. The difference was considered statistically significant at *p* < 0.05. All statistical analysis was performed using IBM SPSS Statistics, version 25 (International Business Machines Corporation, Armonk, NY, USA).

## Results

### Procedures for Generation of a HI Brain Injury Mouse Model

The three ambient temperatures (36.0, 36.5, and 37.0°C) were maintained at the target temperature during hypoxic exposure (Supplementary Figure 1). Body temperatures at the axilla of the HIE mice were measured with a thermometer immediately after exposure to hypoxia; the higher the ambient temperature, the higher was the body temperature of the mice (Supplementary Figure 2).

### Temperature-Dependent Cerebral Volume Loss in the Striatum and Hippocampus Following HI Brain Injury

**Figure 2:**
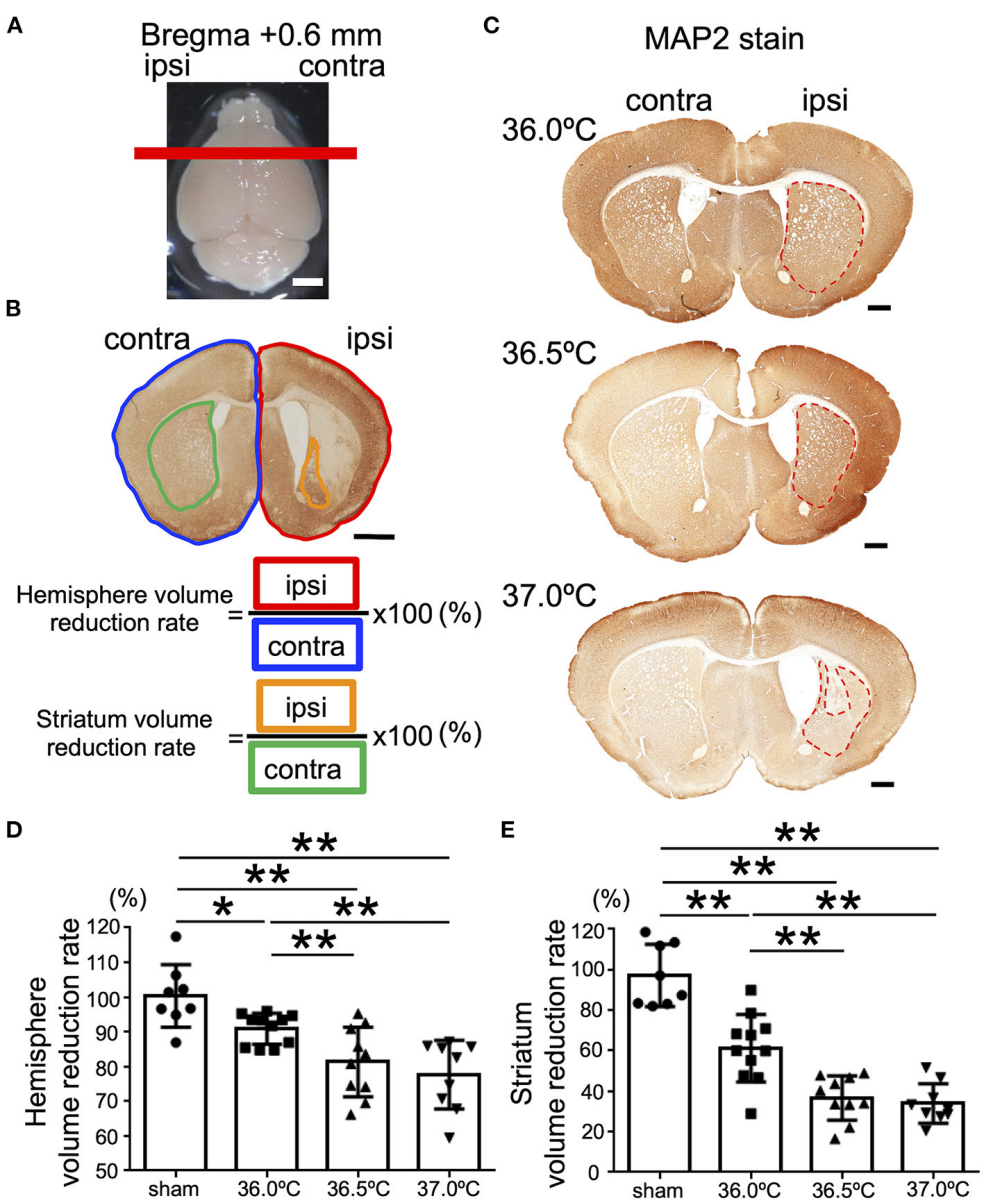
Differences in cerebral volume loss at striatal position due to ambient temperature in HIE mice. **(A)** The red line shows the bregma +0.6 mm, which indicates the position where the brain was cut. Scale bar = 2 mm. **(B)** Calculation method of cerebral volume loss. After MAP2 stain, the whole hemisphere volume reduction rate was calculated by dividing the whole ipsilateral hemisphere (area surrounded by red line) by the whole contralateral hemisphere (area surrounded by blue line). The volume reduction rate of striatum was calculated by dividing the ipsilateral striatum (area surrounded by orange line) by the contralateral striatum (area surrounded by green line). Scale bar = 500 μm. **(C)** MAP2 staining at striatal position 14 days after hypoxia at temperatures of 36.0, 36.5, or 37.0°C. Red dotted line showing MAP2-positive staining areas. Scale bars = 500 μm. **(D,E)** The volume reduction rate at the striatal position in the hemisphere **(D)** or striatum **(E)** (sham, •, *n* = 8; 36.0°C, ■, *n* = 11; 36.5°C, ▲, *n* = 10; 37.0°C, ▼, *n* = 9). ^*^p < 0.05, ^**^p < 0.01. Error bars show means +SD. Ipsi, ipsilateral; contra, contralateral.

The ipsilateral hemisphere of the brain in the 37.0°C group showed volume loss in the contralateral hemisphere (Figure 2A). The difference in volume loss level of brain was investigated following hypoxic exposure at ambient temperatures of 36.0, 36.5, and 37.0°C. At the position of bregma +0.6 mm in the brain section, in which the striatum was observed, the whole ipsilateral brain hemisphere was compared with the contralateral hemisphere (Figure 2A). The areas of the whole ipsilateral brain hemisphere significantly showed volume loss compared with each contralateral side in the 36.0, 36.5, and 37.0°C groups (Figures 2B–D). In addition, the ipsilateral hemisphere decreased in size as the temperature increased (Figure 2D). At the same position of the brain section, the ipsilateral areas of the striatum were observed and compared with the contralateral side of the striatum (Figures 2B,C,E). The striatum was significantly small compared with each contralateral side in the 36.0, 36.5, and 37.0°C groups (Figures 2B,C,E). Furthermore, the ipsilateral striatum size decreased as temperature increased (Figures 2C,E). In a study of several time points (48 h and 1-week), cerebral volume loss gradually became more severe as the ambient temperature increased (Supplementary Figure 3).

**Figure 3:**
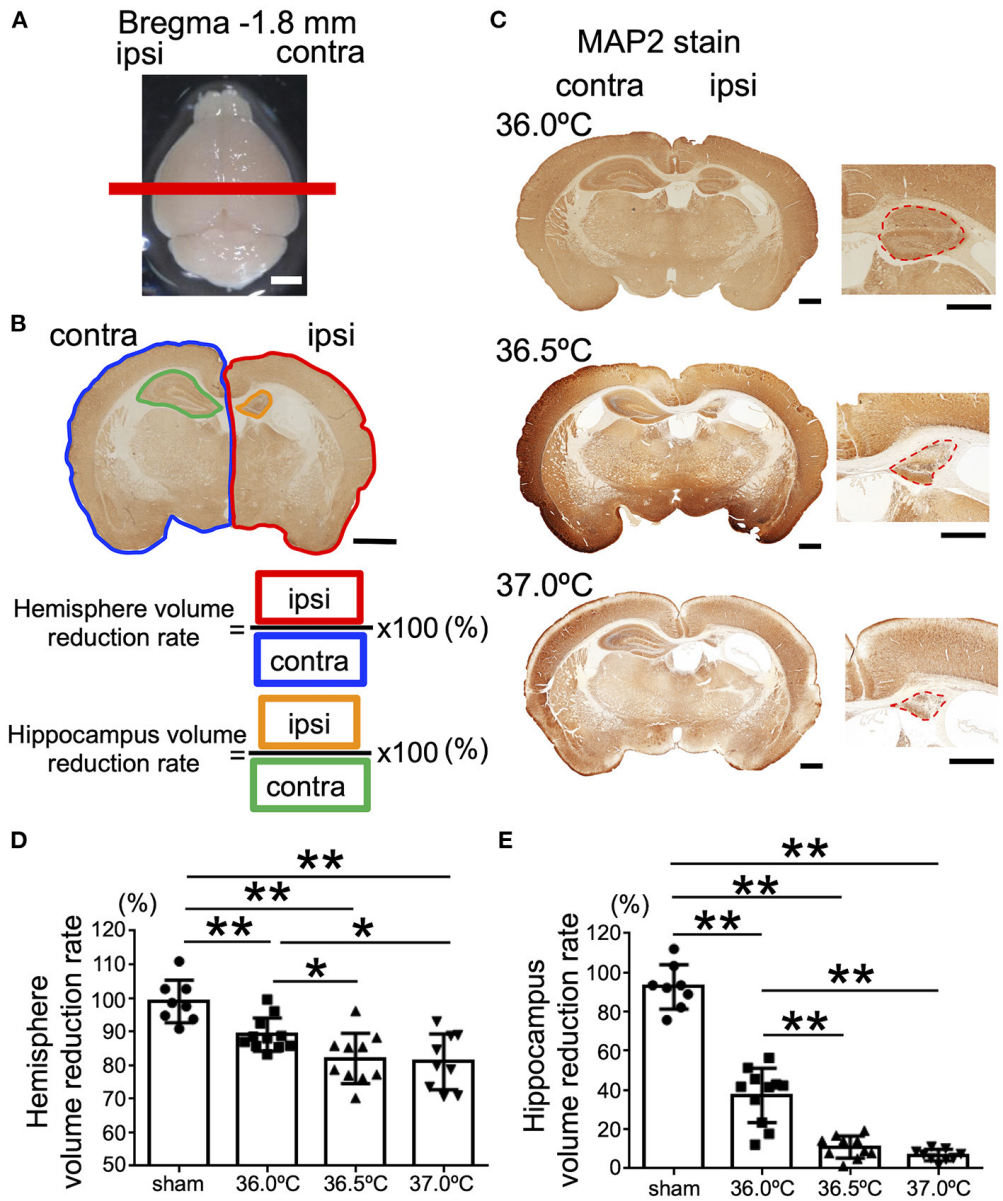
Differences in cerebral volume loss at hippocampal position due to ambient temperature in HIE mice. **(A)** The red line shows the bregma −1.8 mm, which indicates the position where the brain was cut. Scale bar = 2 mm. **(B)** Calculation method of cerebral volume loss. After MAP2 staining, the whole hemisphere volume reduction rate was calculated by dividing the whole ipsilateral hemisphere (area surrounded by red line) by the whole contralateral hemisphere (area surrounded by blue line). The volume reduction rate of the hippocampus was calculated by dividing the ipsilateral hippocampus (area surrounded by orange line) by the contralateral hippocampus (area surrounded by green line). Scale bar = 500 μm. **(C)** MAP2 staining at hippocampal position 14 days after hypoxia at temperatures of 36.0, 36.5, or 37.0°C. Right panels are enlarged images of the hippocampal area in each temperature group. Red dotted lines show MAP2-positive staining areas. Scale bars = 500 μm. **(D,E)** The volume reduction rate at the hippocampal position in the hemisphere **(D)** or hippocampus **(E)** (sham, •, *n* = 8; 36.0°C, ■, *n* = 11; 36.5°C, ▲, *n* = 10; 37.0°C, ▼, *n* = 9). *p < 0.05, **p < 0.01. Error bars show means +SD. Ipsi, ipsilateral; contra, contralateral.

Next, at the position of bregma −1.8 mm in the brain section, in which the hippocampus was observed, the ipsilateral brain hemisphere in the 37.0°C group was compared with the contralateral side (Figure 3A). The areas of the ipsilateral hemisphere were significantly smaller against each contralateral side in the 36.0, 36.5, and 37.0°C groups (Figures 3B–D). In addition, the ipsilateral hemisphere was smaller as the temperature increased, similar to the bregma +0.6 mm position (Figure 3D). At the same position in the brain section, the ipsilateral hippocampus was compared to the contralateral side of the hippocampus at 36.0, 36.5, or 37.0°C (Figures 3B,C,E). The hippocampus was significantly small compared with each contralateral side in the 36.0, 36.5, and 37.0°C groups (Figures 3B,C,E). Like the striatum, the ipsilateral hippocampus size became smaller as the temperature increased (Figures 3C,E). At several time points (48 h and 1-week), cerebral volume loss gradually became more severe as the ambient temperature increased (Supplementary Figure 4).

### Temperature Dependent Accumulation of Microglia in the Striatum and Hippocampus Following HI Brain Injury

**Figure 4:**
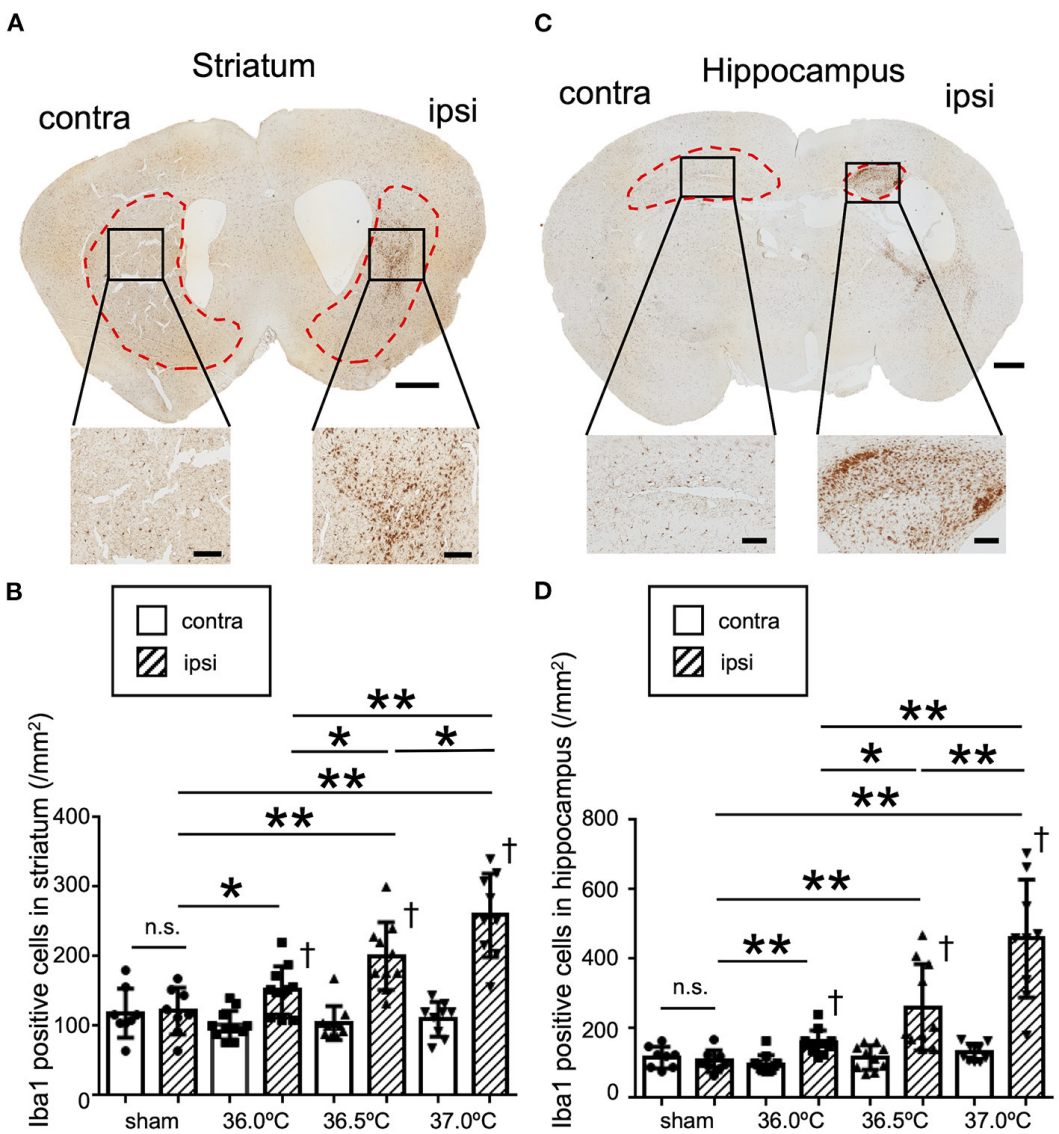
Differences in Iba1 staining due to ambient temperature in HIE mice. **(A)** Iba1 staining in striatal section 14 days after hypoxia at 37.0°C. Red dotted lines show the striatal area. Lower panels show enlarged images of each black square in upper panel. Scale bar = 500 μm in upper panel. Scale bars are 100 μm in lower panels. **(B)** The number of Iba1-positive cells in the ipsi- and contra-lateral striatum 14 days after hypoxia at a temperature of 36.0, 36.5, or 37.0°C. **(C)** Iba1 staining in hippocampal section 14 days after hypoxia at 37.0°C. Red dotted lines show the hippocampal area. Lower panels show enlarged images of each black square in the upper panel. Scale bar = 500 μm in upper panel. Scale bars are 100 μm in lower panels. **(D)** The number of Iba1-positive cells in the ipsi- and contra-lateral hippocampus 14 days after hypoxia at temperatures of 36.0, 36.5, or 37.0°C. (sham, •, *n* = 8; 36.0°C, ■, *n* = 11; 36.5°C, ▲, *n* = 10; 37.0°C, ▼, *n* = 9). *p < 0.05, **p < 0.01 against the ipsi side of another group. ^†^*p* < 0.01 against the contra side. n.s.: not significant. Error bars show means +SD. Ipsi, ipsilateral; contra, contralateral.

To evaluate the difference in the inflammation level in brain lesions among the three different temperatures, Iba1 immunostaining was performed at the bregma +0.6 mm and −1.8 mm positions in the brain sections (Figure 4). In striatum lesions in the bregma +0.6 mm sections, the number of Iba1-positive cells was higher than that on the contralateral side (Figure 4A). The number of Iba1-positive cells in the ipsilateral side gradually increased as the temperature increased; however, that in the contralateral side remained almost same among the control sham and the three HIE groups (Figure 4B). In hippocampal lesions in the bregma −1.8 mm sections, the number of Iba1-positive cells was also higher than in the contralateral side, similar to the striatum (Figure 4C). In the hippocampus and striatum, the number of Iba1-positive cells in the ipsilateral hippocampal lesion gradually increased as the temperature increased, but was almost the same on the contralateral side among all groups (Figure 4D).

### Temperature Dependent Motor Dysfunction Following HI Brain Injury

**Figure 5:**
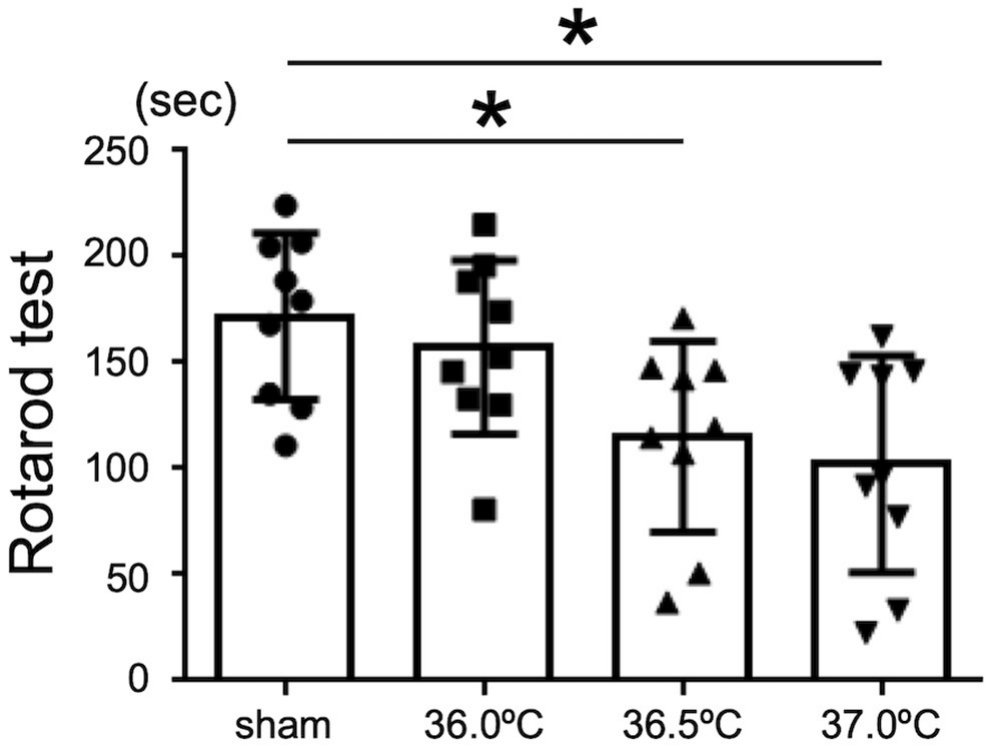
Differences in motor function tests due to ambient temperature in HIE mice. Rotarod test was performed for the evaluation of motor function in HIE mice 2 weeks after hypoxia at temperatures of 36.0°C, 36.5°C, or 37.0°C. (sham, •, *n* = 9; 36.0°C, ■, *n* = 9; 36.5°C, ▲, *n* = 9; 37.0°C, ▼, *n* = 9). *p < 0.05. Error bars show means +SD.

Rotarod tests were performed to evaluate the influence of temperature on motor function in the HIE model mice (Figure 5). Our results showed that the mice performed the test for shorter time in 36.5 and 37.0°C groups compared with the mice in sham group (Figure 5). In 36.5 and 37.0°C groups, motor function was significantly impaired compared with control sham group (Figure 5). However, impairment of motor function was not shown in the 36.0°C group compared with the sham group (Figure 5). The severity of motor function was correlated with the ambient temperature during hypoxia.

### Temperature Dependent Emotional Behavioral Disorder Following HI Brain Injury

**Figure 6:**
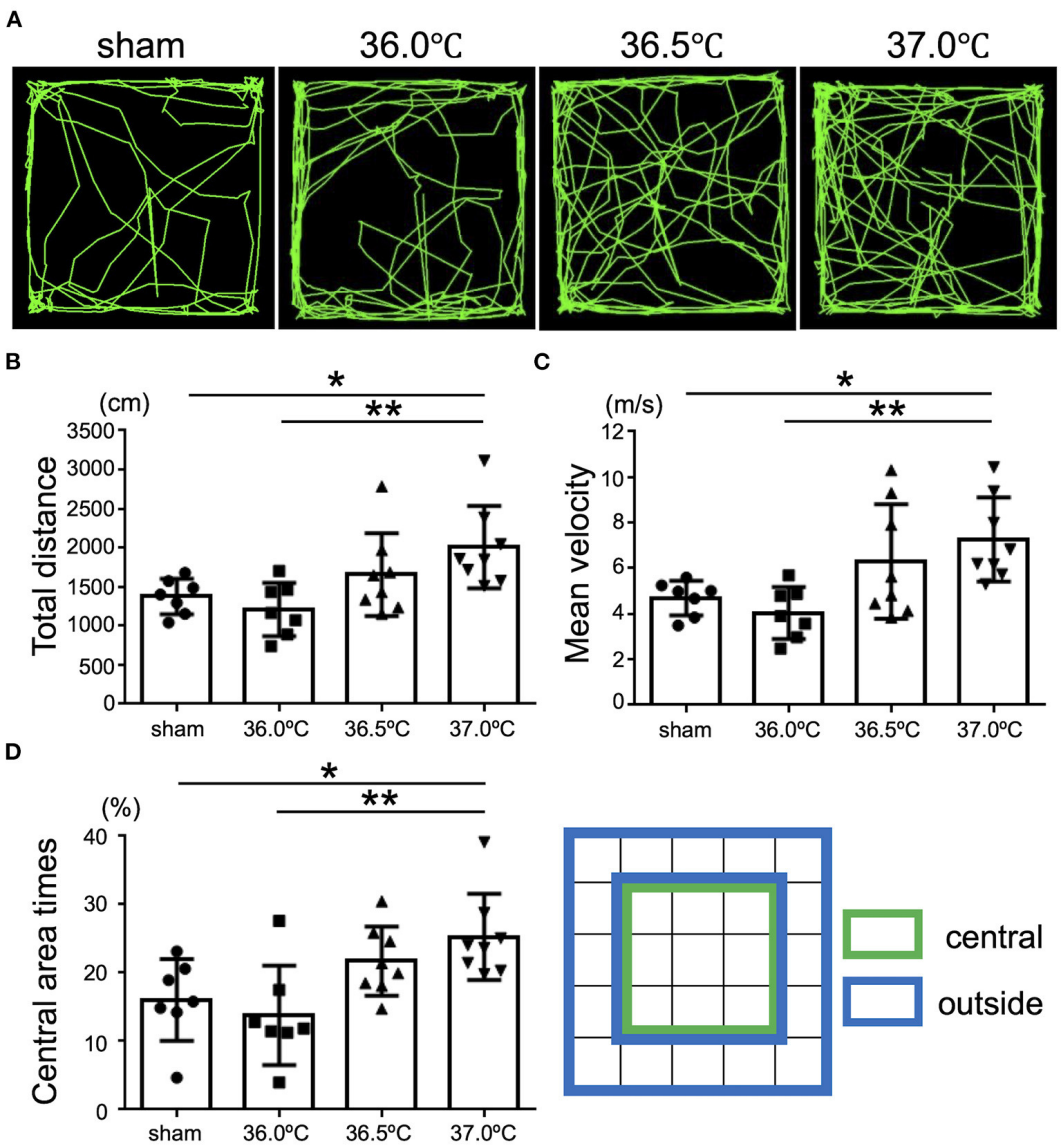
Differences in emotional behavioral dysfunction due to ambient temperature in HIE mice. Open-field tests were performed for evaluation of emotional behavior 2 weeks after hypoxia at temperatures of 36.0°C, 36.5°C, or 37.0°C. **(A)** The representative behavioral trajectory (green line) for 5 min in each temperature group. **(B)** Total distance of walking for 5 min in each temperature group (sham, •, *n* = 7; 36.0°C, ■, *n* = 7; 36.5°C, ▲, *n* = 8; 37.0°C, ▼, *n* = 8). **(C)** Mean speed while walking for 5 min in each temperature group (sham, •, *n* = 7; 36.0°C, ■, *n* = 7; 36.5°C, ▲, *n* = 8; 37.0°C, ▼, *n* = 8). **(D)** The percentage of the time staying in the central area (green) against the outside area (blue) in each temperature group (sham, •, *n* = 7; 36.0°C, ■, *n* = 7; 36.5°C, ▲, *n* = 8; 37.0°C, ▼, *n* = 8). *p < 0.05, **p < 0.01. Error bars show means +SD.

To evaluate higher brain function, open-field tests were performed to investigate the influence of temperature on emotional behavior in the HIE model mice (Figure 6). The behavior of the HIE mice was observed in the open-field area for 5 min and their behavior trajectories were recorded (Figure 6A). In the 37.0°C group, the total distance of movement was significantly longer than that in the sham and 36.0°C groups (Figure 6B). In the 37.0°C group, the mean velocity of movement was significantly higher compared with that in the sham and 36.0°C groups (Figure 6C). From the above results, mice in the 37.0°C group showed hyperactivity and restlessness. Next, we investigated how long the mice stayed in the central area (green area in Figure 6D, right panel) of the open field during the open-field test (Figure 6D). As shown in the trajectory lines of the 37.0°C group in Figure 6A, mice in the 37.0°C group moved around a larger portion of the field compared with the sham and 36.0°C groups, and remained in the central area longer than mice in the sham and 36.0°C groups (Figures 6A,D). These results indicated that high temperature during the generation of HIE model mice induced greater brain impairment, resulting in behavioral deficits such as hyperactivity, which is known to be an abnormal emotional behavior. The severity of the anxious behavior correlated with ambient temperature during hypoxia.

### Summary of Cerebral Volume Loss and Behavioral Disorder in HIE Mouse Model due to Ambient Temperature During Exposure to Hypoxia

**Figure 7:**
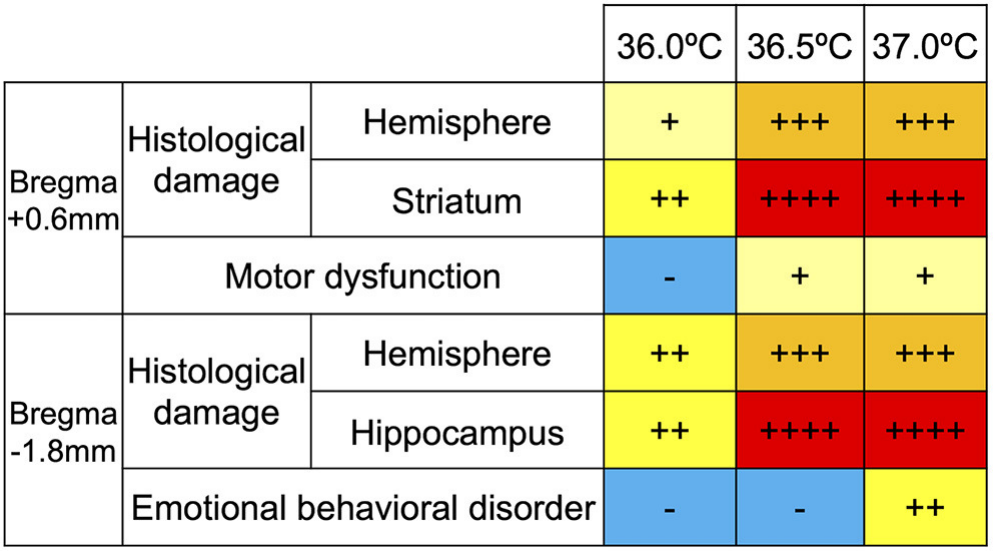
Grade of temperature dependent cerebral volume loss and behavioral dysfunction. The histological damage to the striatum and hippocampus were expressed in four grades (+, ++, +++ and ++ ++) in each temperature group. The levels of disturbance in motor function and emotional behavior were classified into three grades (–, +, and ++) in each temperature group.

The degree of histological change and behavioral disorder was classified according to severity, based on the data of this study, and the degree was compared among the ambient temperatures. In addition, to evaluate the link between the histological and behavioral findings, correlations between the following are summarized (Figure 7): motor function (assessed using the rotarod tests) and striatal lesions, considered to be part of the causative lesion; emotional behavioral disorder (assessed using the open-field tests) and hippocampal lesions, considered to be part of the causative lesion. According to our grading of histological severity in HIE mice, the high temperature groups showed more damage, especially in the striatum, and the hippocampus showed severe cerebral volume loss (Figure 7). In behavioral evaluation, neither motor dysfunction nor emotional behavioral disorder were observed in the 36.0°C groups. In contrast, both behavioral abnormalities were observed in the 37.0°C group (Figure 7). The severity of these behavioral abnormalities was consistent with the grade of the histological damage. In summary, the higher the ambient temperature, the more severe is the damage to the HIE model. The group at 36.0°C was a moderate model, which had only histological damage, and the group at 37.0°C was a severe model, which had not only histological damage but also behavioral abnormalities.

## Discussion

This study is the first to investigate the relationship between a gradual slight change in ambient temperature during hypoxic exposure and the severity of pathology and behavioral impairments in HIE. We report that even a small change of 0.5°C showed a significant difference in pathological and behavioral changes in a neonatal mouse model of HIE. In addition, there was a correlation between ambient temperature and microglial accumulation.

It has been reported that HIE is exacerbated when the ambient temperature during hypoxic exposure is set to a high temperature, such as 40 or 42°C (13, 14). In contrast, hypothermia therapy at 33 or 34°C after hypoxia is effective for treating HIE, which is consistent with the severity of brain injury being affected by the ambient temperature (1, 6, 25, 29). However, the influence of the slight change in the ambient temperature during hypoxia from 36 to 37°C, which is the physiological range of normal intrauterine temperature, has not yet been clear. We set the ambient temperature to 36.0, 36.5, or 37.0°C during hypoxia to clarify the effect of ambient temperature. In this study, the higher the temperature during hypoxia, the more severe was the brain volume reduction rate and behavioral dysfunction. There is a correlation between ambient temperature, cerebral volume loss, and behavioral dysfunction in a neonatal mouse model of HI brain injury. Even a small change of 0.5°C produced a significant difference in pathological and behavioral changes.

In detailed pathological findings, volume loss of the hippocampus was stronger than that of the striatum in the HIE model. Corresponding to these pathological changes, emotional behavior also showed severe dysfunction. This suggests that different brain regions react differently to hypoxia. This is consistent with the vulnerability of the hippocampus to hypoxia owing to the lack of vascular territory (30, 31).

Previous studies did not consider ambient temperature because it was difficult to fully control the temperature of the hypoxic chamber. We used a special chamber with a heater raiser plate that has exhaust ducts and fans to circulate the inside air, which can tightly and automatically control the ambient temperature. As the chamber we used was excellent at maintaining the temperature inside, the axillary temperature of mice after HIE was correlated with ambient temperature. In addition, we carefully considered the position of the pups during hypoxia to reduce the difference in body temperature after HIE. As a result, we could clearly observe temperature-dependent brain injuries.

Microglia are the primary immune cells within the brain (3, 32), and are reported to accumulate following HIE damage (3, 19, 33). Similar to previous reports, microglia were observed more commonly in the ipsilateral damaged brain than in the contralateral side in this study. In addition, the accumulation of microglia following HI brain injury has been shown to be much dependent on ambient temperature. There is a correlation between ambient temperature and microglial accumulation; the higher the ambient temperature, the stronger the inflammation. Even a small change of 0.5°C produced a significant difference in the accumulation of microglia, which was correlated with the severity of the HIE model. These results suggest that microglia are deeply involved in the severity of HIE due to changes in the ambient temperature. Therefore, control of microglial inflammation is expected to lead to the development of novel therapies for HIE.

To evaluate the difference in inflammation levels, Iba1 immunostaining was performed in this study. The origin of the Iba1-positive cells is unclear. Previous studies have shown that microglial activation in HIE was derived from both resident microglia and infiltrating blood-derived macrophages (33). It is also known that microglia exhibit pro-inflammatory or anti-inflammatory phenotypes depending on the condition and timing of the neuroinflammation state (34–36). Further investigation of microglial activation is required to determine the inflammation associated with HIE.

Considering that factors other than ambient temperature affect HIE severity, it should be noted that all steps of HIE generation had the potential to affect it. Both unilateral CCA ligation and hypoxic exposure are widely used methods for producing HIE mice models (2, 37). However, the grade of brain injury and symptomatic phenotype caused by HIE have been reported to be different individually (2, 12, 38) because the tissues of neonatal pups are immature and have individual differences in growth and variability of intracranial artery (16, 39–42). In addition, differences between male and female exist in outcomes of HIE mice models (36, 43). We also used both P9 and P10 mice as experimental limitation. It may be better to be used exact same-day-old mice because neonatal pups develop rapidly in 1 day both physically and neurologically. However, quite meaningful results were obtained despite using P9-10 mice, which may suggest the ambient temperature affects much more than the 1-day growth. In contrast, as previously reported, we should focus not only on differences of animals but also on the composition of the procedures during hypoxic exposure as oxygen concentration and exposure time (38, 44, 45). By strictly controlling the ambient temperature, severity could be reproduced at various levels as same as the arrangement of oxygen concentration and hypoxic exposure time. Therefore, our findings suggested that it is important to maintain the ambient temperature to which the mice are exposed. Here, we succeeded to reproduce the HIE mice from mild model which had only histological damage to severe model which had not only histological damage but also behavioral abnormalities by adjusting the ambient temperature. From a clinical point of view, the ambient temperature could be supposed as the intrauterine temperature of the mother. Therefore, it is suggested that if the mother's body temperature is high, the degree of damage may be stronger even in the same oxygen concentration and hypoxic exposure time. Thus, experimental models described in this report are worthy of further HIE experimental systems.

In this study, even a small change of 0.5°C produced a significant difference in pathology and behavior in a neonatal mouse model of HI brain injury. Additionally, there was a correlation between ambient temperature and microglial accumulation, which is expected to lead to the development of novel therapies for HIE. Tight control of ambient temperature is useful for creating a rodent model with appropriate severity.

## Data Availability Statement

The original contributions presented in the study are included in the article/Supplementary Material, further inquiries can be directed to the corresponding author/s.

## Ethics Statement

The animal study was reviewed and approved by the Institutional Animal Care and Usage Committee of Shiga University of Medical Science.

## Author Contributions

RZ conducted the experiments, analyzed the data, and drafted the manuscript. TT designed the study, provided advice on the experimental procedures, and assisted with the writing and revision of the manuscript. ST provided advice on the experimental procedures and revised the manuscript. YN and AH secured funding and supported the experiments. HKa provided the advice on emotional behavior test. HKo, TM, MK, and NO provided the advice on techniques, expertise, and feedback. All authors have read and approved the final manuscript.

## Funding

This study was supported by JSPS KAKENHI Grant Numbers JP20K16922 and JP20K18165 and by the Ogyaa Donation Foundation from the Japan Association of Obstetricians and Gynecologists.

## Conflict of Interest

The authors declare that the research was conducted in the absence of any commercial or financial relationships that could be construed as a potential conflict of interest.

## Publisher's Note

All claims expressed in this article are solely those of the authors and do not necessarily represent those of their affiliated organizations, or those of the publisher, the editors and the reviewers. Any product that may be evaluated in this article, or claim that may be made by its manufacturer, is not guaranteed or endorsed by the publisher.

## Abbreviations

CCA: common carotid artery
HI: hypoxic-ischemic
HIE: hypoxic-ischemic encephalopathy.
