# Supplementary material for: Ambient Temperature Is Correlated With the Severity of Neonatal Hypoxic-Ischemic Brain Injury via Microglial Accumulation in Mice
Source: Front Pediatr. 2022 May 6;10:883556. doi: 10.3389/fped.2022.883556 (PMC9120824; doi:10.3389/fped.2022.883556)
Supplement: Supplementary file 1 [file Data_Sheet_1.PDF]

Supplementary Figure 1

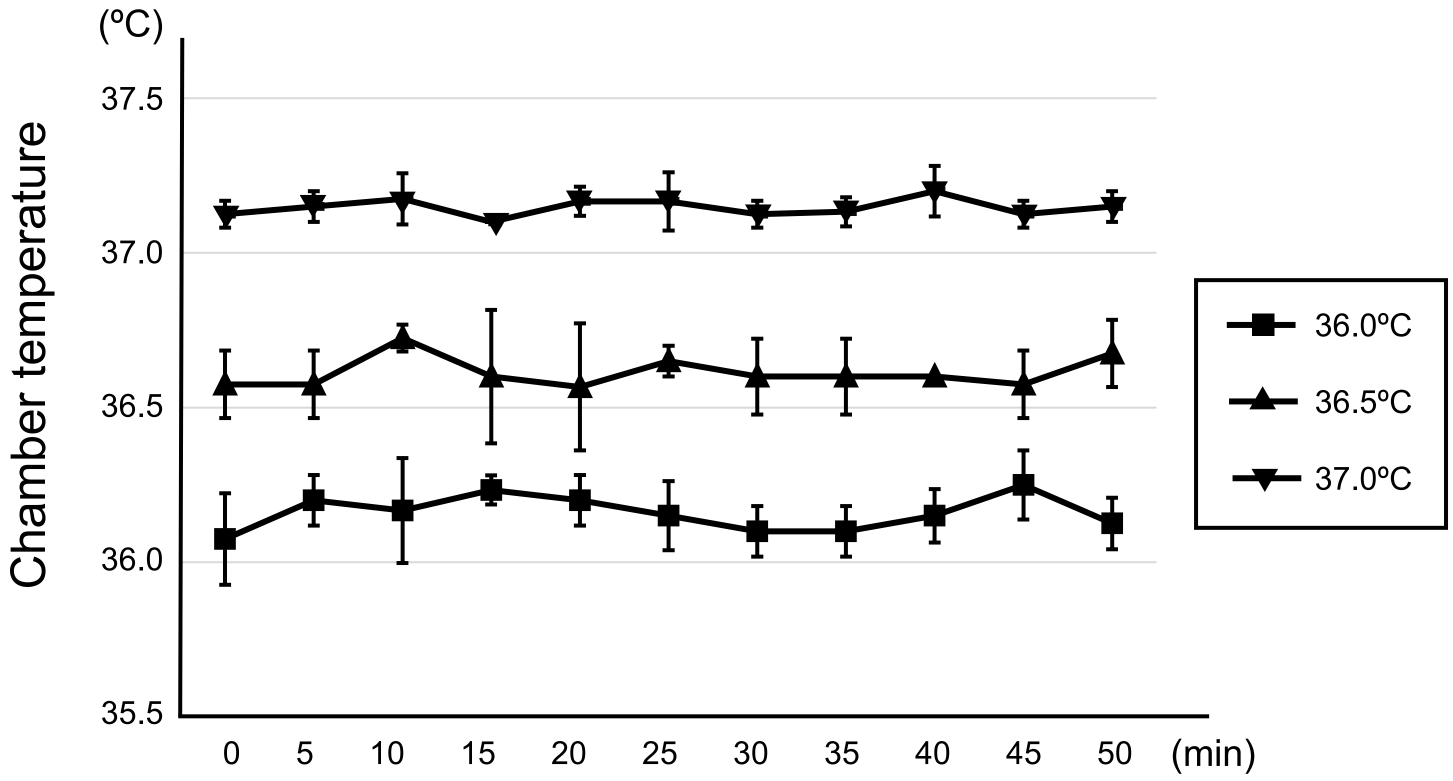

Supplementary Figure 1. Temperature changes in the hypoxic chamber adjusted to 36.0 °C, 36.5 °C, or 37.0 °C during hypoxic exposure. Line graphs showing the measured temperatures inside the chamber while setting the target temperature to 36.0 °C (■, n=4), 36.5 °C (▲, n=4) or 37.0 °C (▼, n=4). Ambient temperatures were recorded every 5 min from 0 to 50 min during hypoxia. Error bars show means ± SD.

Supplementary Figure 2

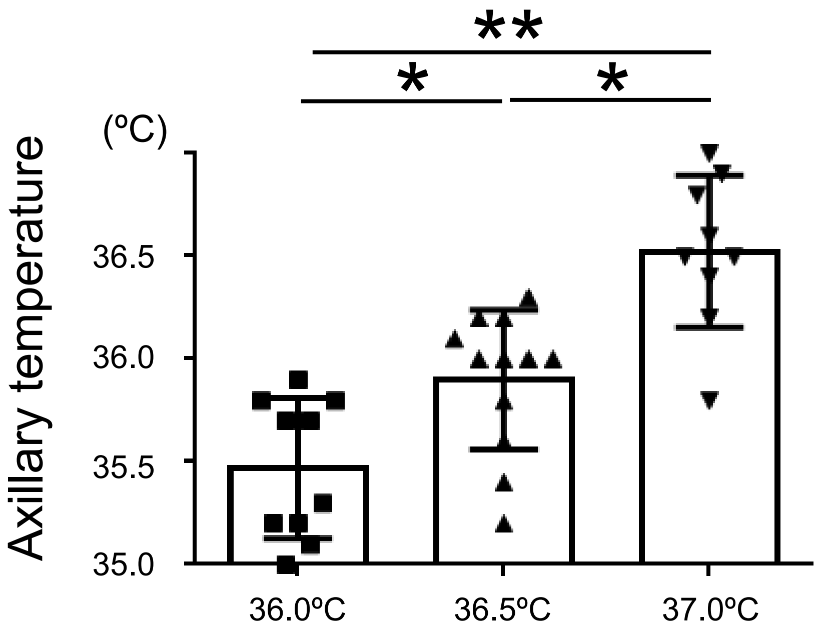

Supplementary Figure 2. Axillary temperature immediately after hypoxia in HIE mice. Graphs showing the axillary temperatures measured immediately after hypoxia with the target ambient temperatures set to 36.0 °C (■, n=10), 36.5 °C (▲, n=12) , or 37.0 °C (▼, n=9). *p <0.05, **p <0.01. Error bars show means +SD.

Supplementary Figure 3

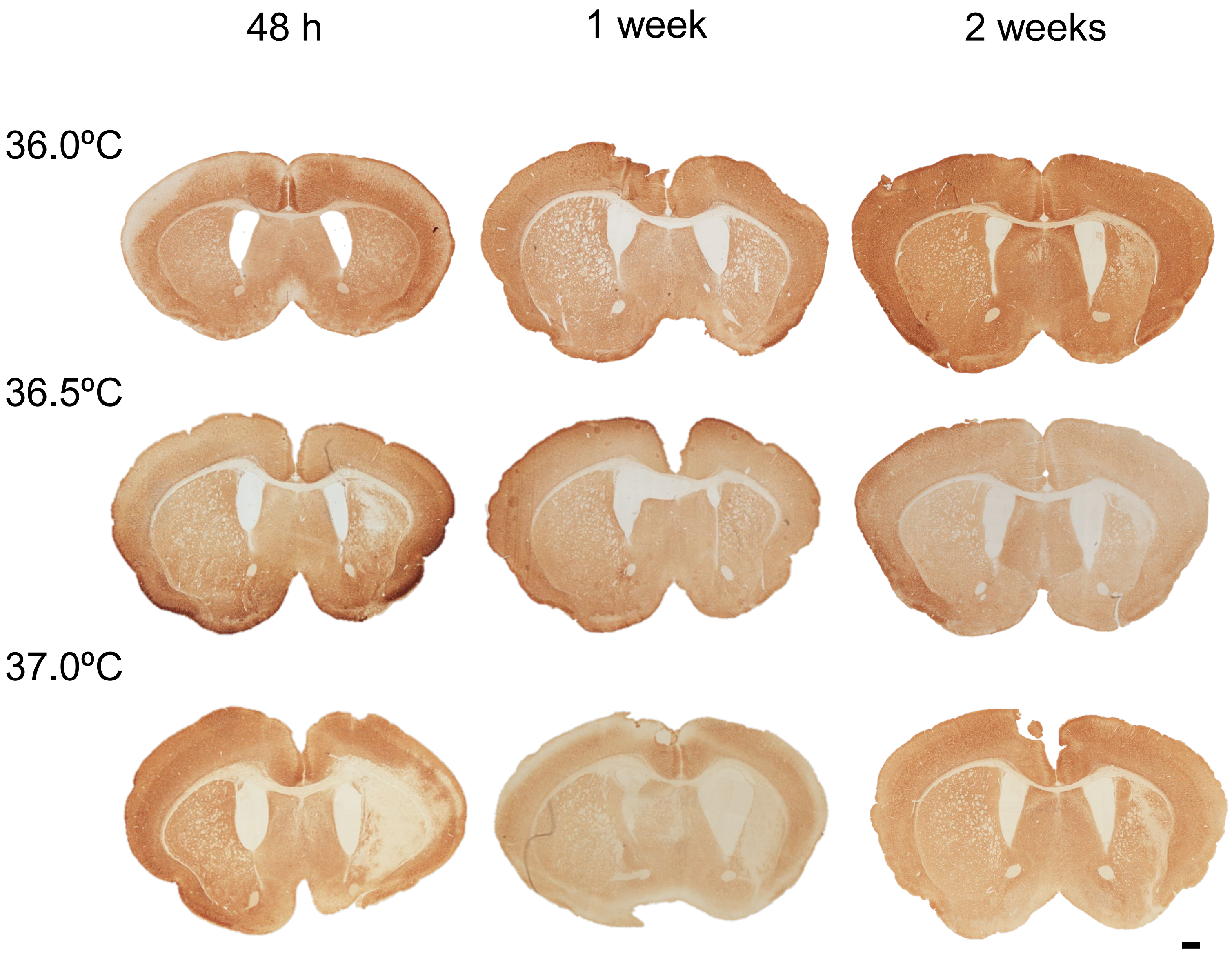

Supplementary Figure 3. Differences in brain atrophy at striatal position at different time courses among three ambient temperatures of HIE mice. Pictures showing MAP2 staining at striatal position 48 h, 1 week, and 2 weeks after hypoxia at temperatures of 36.0 °C, 36.5 °C, or 37.0 °C. Scale bar = 500 μm.

Supplementary Figure 4

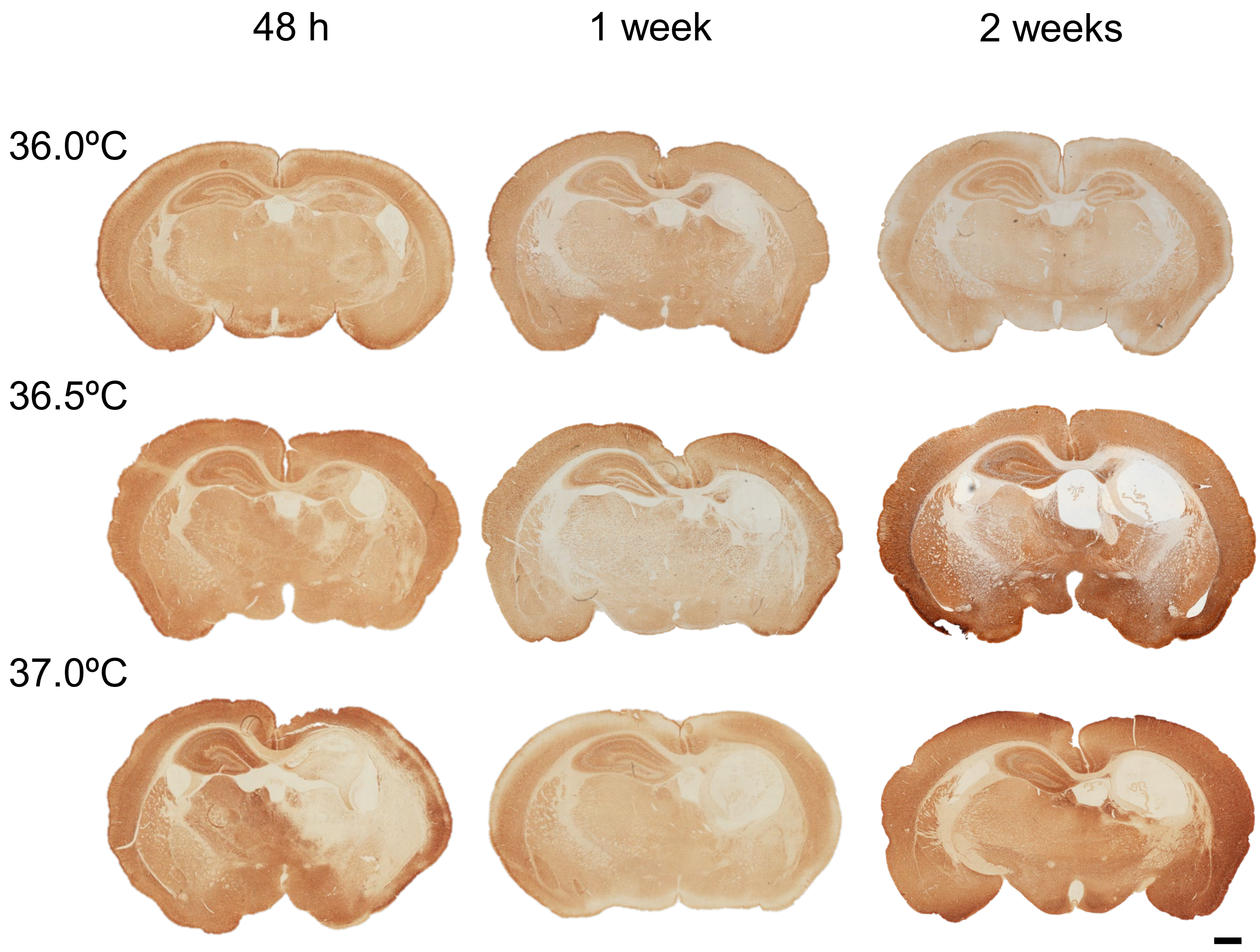

Supplementary Figure 4. Differences in brain atrophy at hippocampal position at several time courses among three ambient temperatures of HIE mice. Pictures showing MAP2 staining at hippocampal position 48 h, 1 week, and 2 weeks after hypoxia at temperatures of 36.0 °C, 36.5 °C, or 37.0 °C. Scale bar = 500 µm.
